# Critical Role of Flavin and Glutathione in Complex I–Mediated Bioenergetic Failure in Brain Ischemia/Reperfusion Injury

**DOI:** 10.1161/STROKEAHA.117.019687

**Published:** 2018-04-11

**Authors:** Anja Kahl, Anna Stepanova, Csaba Konrad, Corey Anderson, Giovanni Manfredi, Ping Zhou, Costantino Iadecola, Alexander Galkin

**Affiliations:** 1 ^1^From the Feil Family Brain and Mind Research Institute, Weill Cornell Medicine, New York, NY (A.K., A.S., C.K., C.A., G.M., P.Z., C.I., A.G.); 2 ^2^School of Biological Sciences, Queen’s University Belfast, United Kingdom (A.S., A.G.)

**Keywords:** flavin mononucleotide, glutathione, mitochondria, oxidative stress, reperfusion

## Abstract

**Background and Purpose—:**

Ischemic brain injury is characterized by 2 temporally distinct but interrelated phases: ischemia (primary energy failure) and reperfusion (secondary energy failure). Loss of cerebral blood flow leads to decreased oxygen levels and energy crisis in the ischemic area, initiating a sequence of pathophysiological events that after reoxygenation lead to ischemia/reperfusion (I/R) brain damage. Mitochondrial impairment and oxidative stress are known to be early events in I/R injury. However, the biochemical mechanisms of mitochondria damage in I/R are not completely understood.

**Methods—:**

We used a mouse model of transient focal cerebral ischemia to investigate acute I/R-induced changes of mitochondrial function, focusing on mechanisms of primary and secondary energy failure.

**Results—:**

Ischemia induced a reversible loss of flavin mononucleotide from mitochondrial complex I leading to a transient decrease in its enzymatic activity, which is rapidly reversed on reoxygenation. Reestablishing blood flow led to a reversible oxidative modification of mitochondrial complex I thiol residues and inhibition of the enzyme. Administration of glutathione-ethyl ester at the onset of reperfusion prevented the decline of complex I activity and was associated with smaller infarct size and improved neurological outcome, suggesting that decreased oxidation of complex I thiols during I/R-induced oxidative stress may contribute to the neuroprotective effect of glutathione ester.

**Conclusions—:**

Our results unveil a key role of mitochondrial complex I in the development of I/R brain injury and provide the mechanistic basis for the well-established mitochondrial dysfunction caused by I/R. Targeting the functional integrity of complex I in the early phase of reperfusion may provide a novel therapeutic strategy to prevent tissue injury after stroke.

## Abstract 2

Supplemental Digital Content is available in the text.

Stroke remains a leading cause of death and disability worldwide.^1^ Despite decades of research, tissue-type plasminogen activator and endovascular devices are the only available treatment options.^2^ However, because of a narrow therapeutic time window and potential contraindications, only 3% to 5% of stroke patients are able to benefit from these interventions.^3^ This highlights the need for a broader understanding of tissue injury mechanisms to develop more effective treatments.

The loss of cerebral blood flow leads to decreased oxygen levels, impairment of mitochondrial oxidative phosphorylation and energy failure in the ischemic area, initiating a sequence of pathophysiological events that after reoxygenation lead to ischemia/reperfusion (I/R) damage.^4^ Mitochondria play a key role in ischemic brain injury, both through impairment of mitochondrial ATP production with bioenergetic dysfunction and oxidative stress and by mediating cell death pathways.^5,6^ The lack of oxygen resulting from ischemia leads to impaired mitochondrial ATP production (primary energy failure), collapse of the mitochondrial membrane potential, and, consequently, activation of intrinsic cell death pathways.^7,8^

After reperfusion, there is a transient restoration of bioenergetic state, which is followed by a second phase of energy depletion (secondary energy failure) leading to delayed tissue damage.^9,10^ This sequence of events has been confirmed by several laboratories, which have also ruled out microcirculatory failure or changes in substrate availability as the cause of the secondary energy depletion and cell death.^11,12^ In fact, data indicate that secondary energy failure after transient ischemia might be the result of delayed mitochondrial damage, likely because of oxidative stress.^11,12^ Mitochondrial electron transport chain (ETC) enzymes are known to become rapidly over-reduced in the absence of oxygen and to be damaged by subsequent reoxygenation.^11,13,14^ However, despite intensive research, the molecular mechanisms of mitochondria damage in I/R remain to be elucidated.

Here, we used a mouse model of middle cerebral artery occlusion (MCAO) to investigate acute I/R-induced changes of mitochondrial function, focusing on the molecular and biochemical mechanisms of primary and secondary energy failure. Our results suggest a central role of mitochondrial complex I (C-I) impairment in the development of bioenergetic failure after acute I/R brain injury. Protection of C-I enzymatic function during ischemia and the initial stages of reperfusion could be an effective approach to prevent subsequent detrimental events in the I/R cascade, ultimately preserving neuronal integrity and reducing brain damage after stroke.

## Materials and Methods

All data and materials have been made publicly available at the https://pure.qub.ac.uk/portal/ repository, and a detailed Methods section is available in the online-only Data Supplement.

### MCAO Model

All procedures were approved by the Institutional Animal Care and Use Committee of Weill Cornell Medicine and performed in accordance with the ARRIVE guidelines (Animals in Research: Reporting In Vivo Experiment).^15^ Transient MCAO was induced using an intraluminal filament as described.^16^ In brief, 7- to 9-week-old, male mice were anesthetized with 1.5% to 2.0% isoflurane and rectal temperature was maintained at 37.3±0.3°C. Cerebral blood flow was measured with laser-Doppler flowmetry (Periflux System 5010; Perimed) in the ischemic center (2 mm posterior, 5 mm lateral to bregma). After 35 minutes, the filament was retracted and cerebral blood flow reestablished. This duration of cerebral ischemia has been used extensively by us^17,18^ and others^19^ and leads to reproducible infarct volumes of 50 to 60 mm^3^ and measurable neurological deficits. Only animals that exhibited a reduction in cerebral blood flow 85% during MCAO and in which cerebral blood flow recovered by 80% after 10 minutes of reperfusion were included in the study.^20,21^ Three days after, MCAO functional impairment was assessed and infarct volume was quantified in cresyl violet–stained sections and corrected for swelling, as previously described.^16^

### Administration of Glutathione-Ester and Glutathione Content Measurement

Reduced glutathione-ethyl ester (G1404; Sigma Aldrich) was administered immediately after the initiation of reperfusion via jugular vein (400 mg/kg). Saline injections served as control. Total glutathione content was determined using Glutathione Assay Kit (703002; Cayman).

### Mitochondrial Measurements

After MCAO alone or MCAO with a period of recirculation as indicated, mice were decapitated. Brains were removed and a standardized 4 mm MCA area tissue sample dissected using a mouse brain matrix (Zivic Instruments). The brain sample was homogenized in ice-cold isolation buffer (in mmol/L: 210 mannitol, 70 sucrose, 1 ethylene glycol-bis(β-aminoethyl ether)-N,N,N’,N’-tetraacetic acid, 5 HEPES, pH 7.4) with 80 strokes of a Dounce homogenizer. The homogenate was centrifuged at 1000*g* for 5 minutes at 4°C and the supernatant was collected and used for respiration analysis. Respiration was measured using Oxygraph-2k (Oroboros Instruments).

For isolation of mitochondria, brain homogenates were centrifuged for 15 minutes at 20 000*g*. The obtained membrane pellet was rinsed twice with (in mmol/L): 250 sucrose, 50 Tris-HCl (pH 7.5), 0.2 EDTA medium, and subsequently resuspended in the same medium. Frozen aliquots were stored at −80°C until use. Protein content was determined by bicinchoninic acid assay (Sigma) with 0.1% deoxycholate for solubilization of mitochondrial membranes.

### Mitochondria and Respiratory Chain Analysis

Activities of respiratory chain enzyme and citrate synthase were measured spectrophotometrically as described.^22^ Flavin mononucleotide (FMN) was determined fluorometrically.^23^ Immunoblot analyses were performed using OXPHOS antibody cocktail (ab110413; Abcam).^22^

### Experimental Design and Statistical Analysis

Mice were randomly assigned to the experimental groups, and analyses were performed by an investigator blinded to the treatment protocol. Data are expressed as mean±SEM. Differences were considered statistically significant when *P<0.05. Details of statistical analyses are indicated in the Figure legends and online-only Data Supplement.

## Results

### Multiphasic Impairment of Mitochondrial Respiration in I/R

**Figure 1.**
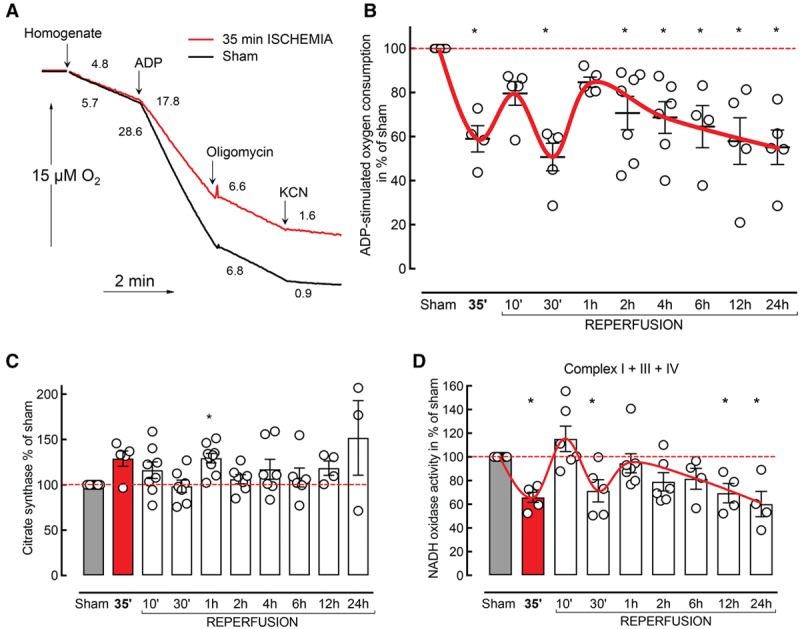
Multiphasic pattern of mitochondrial respiratory decline after ischemia/reperfusion (I/R). **A**, Representative traces of mitochondrial oxygen consumption from sham (red) and 35 minute ischemia (black) in whole tissue homogenates. Addition of 1 mM cyanide (KCN) almost fully inhibited respiration in homogenates. **B**, Effect of I/R injury on ADP-stimulated oxygen consumption (sham: n=19; different time points n as indicated by the dots; Kruskal–Wallis test with Dunn multiple comparisons test; **C**) citrate synthase (**C**) and NADH oxidase (**D**) activity in the same preparations.

We studied I/R-induced changes of mitochondrial function in a mouse model of focal ischemia after transient MCAO. Figure 1A shows representative traces of malate/glutamate-supported respiration of brain homogenates of sham and after 35 minutes ischemia. ADP-stimulated mitochondrial respiration showed multiphasic impairment after I/R (Figure 1B). A decline (59.0±5.9% of sham control; *P*<0.05; n=4 per group) was observed during ischemia, followed by a partial recovery (79.6±5.4% of control; *P*>0.05; n=5 per group) at 10 minutes of reperfusion, and by a subsequent profound decline in respiration (50.7±6.2% of control; *P*<0.05; n=5 per group) at 30 minutes of reperfusion. These early changes in mitochondrial function were followed by a recovery of respiration at 1 hour of reperfusion (84.7±2.3% of control; *P*>0.05; n=5 per group) and then by a progressive decline in respiration, occurring 2 to 24 hours (55±7.8% of control; *P*<0.05; n=5 per group, at 24 hours) after reperfusion (Figure 1B).

Citrate synthase activity, an indicator of mitochondrial content, did not significantly differ from control at any time point (*P*>0.05; n=3–7 per group; Figure 1C). Further, we did not detect significant changes in the protein levels of ETC complexes I to V and mitochondrial respiratory control ratio, during the ischemic phase or within 24 hours after reperfusion (Figure I in the online-only Data Supplement).

### Activities of Individual Mitochondrial Membrane Complexes Are Differently Affected in I/R

We note that brain homogenates included nonsynaptic mitochondria but also synaptosomes containing synaptic mitochondria. The synaptic mitochondria, however, do not contribute to ADP-stimulated respiration, because of restricted ADP access to synaptosomes. Respiration measured in whole tissue homogenates is a product of several processes including transport of substrates, activities of NAD-dependent dehydrogenases, and ETC.

For ETC activity measurements, substrate delivery into all mitochondrial populations was ensured by addition of the membrane-permeabilizing agent alamethicin. To specifically assay for I/R-induced changes in ETC complexes, we assessed the overall activity of the respiratory chain by measuring NADH oxidase (complexes I+III+IV; Figure 1D). The temporal profile of NADH oxidase activity changes strongly corresponded to the multiphasic pattern observed for the mitochondrial respiration (Figure 1B), suggesting that the observed mitochondrial dysfunction is a result of I/R-induced ETC impairment.

**Figure 2.**
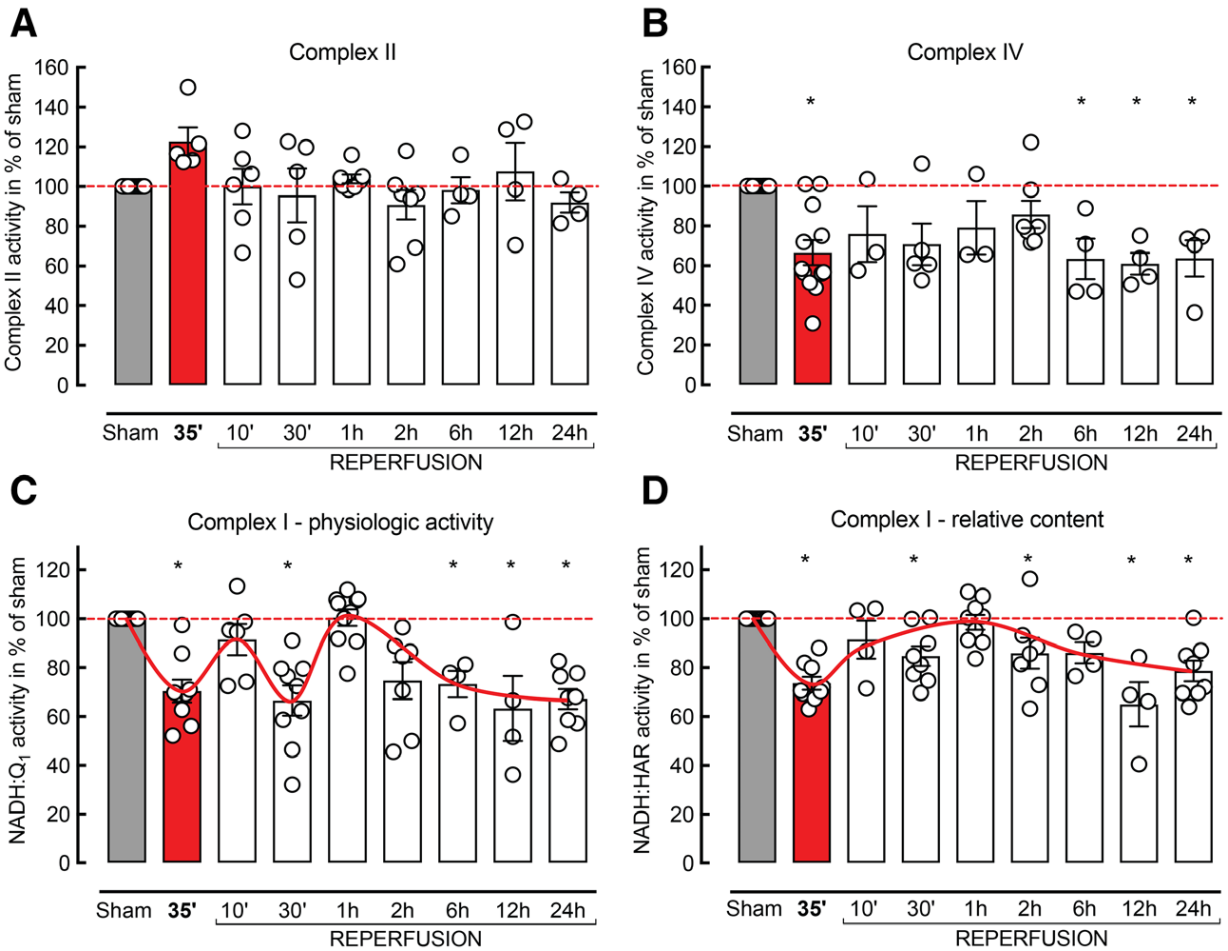
Enzymatic activities of respiratory chain complexes are differently affected after ischemia/reperfusion (I/R). **A**, Complex II (C-II), (**B**) C-IV, and (**C**) C-I NADH:Q_1_ and (**D**) C-I NADH:hexaammineruthenium (HAR) reductase activities were measured in whole tissue homogenates; n=4 to 12 per group; Kruskal–Wallis test.

Next, we measured the activities of individual ETC complexes: succinate dehydrogenase (C-II), ferrocytochrome *c* oxidase (C-IV), and NADH:ubiquinone oxidoreductase (C-I), as well as succinate:cytochrome *c* reductase (C-II+C-III). C-II–linked activities were not affected at any time point after I/R, indicating that C-II and C-III were not responsible for the I/R-induced mitochondrial dysfunction (*P*>0.05; n=4–7 per group; Figure 2A; C-II+III data not shown).

C-IV activity was lower at all time points compared with sham (63.7±9.1% at 24 hours; *P*<0.05; n=4; Figure 2B). However, the decline of C-IV did not follow the multiphasic pattern observed in ADP-stimulated respiration and NADH oxidase activity, suggesting that the mechanism of C-IV and NADH oxidase impairment is different and that C-IV is not responsible for the I/R-induced changes.

### Complex I Impairment Is Associated With the Multiphasic Pattern of Respiratory Decline in I/R

To elucidate the mechanisms of C-I impairment, we measured the physiological activity and the relative amount of C-I using two different approaches. The physiological activity of C-I was assessed as NADH:Q_1_ reductase. The relative content of C-I (proportional to flavin [FMN] content in the enzyme) was determined as oxidation of NADH by hexaammineruthenium (HAR). The NADH:HAR reaction occurs only at the head of the enzyme, where HAR accepts electrons from the FMN, the first redox center of C-I.^24^

The physiological activity of C-I (Figure 2C) followed the same pattern as ADP-stimulated respiration (Figure 1B) and NADH oxidase activity (Figure 1D), indicating that the impairment of oxidative phosphorylation in the ischemic tissue was because of a specific dysfunction of C-I. Interestingly, NADH:HAR reductase showed an apparent decrease in the relative content of C-I after 35 minutes of ischemia (68.7±1.3%; *P*=0.0001; n=6 per group), followed by a rapid recovery after reoxygenation (97.0±3.9%; *P*=0.99; n=6 per group) and a slow gradual decline at subsequent time points after I/R injury (78.7±4.3%; *P*=0.0003; n=8 per group; Figure 2D).

On the basis of the results above, we identified 35 minutes ischemia and 30 minutes, 1 hour, and 24 hours of reperfusion as critical time points for the development of mitochondrial dysfunction in I/R injury. To further elucidate the mechanism of C-I impairment, we assayed NADH:HAR and NADH:Q_1_ reductase activity in preparations of mitochondrial membranes isolated at these time points.

**Figure 3.**
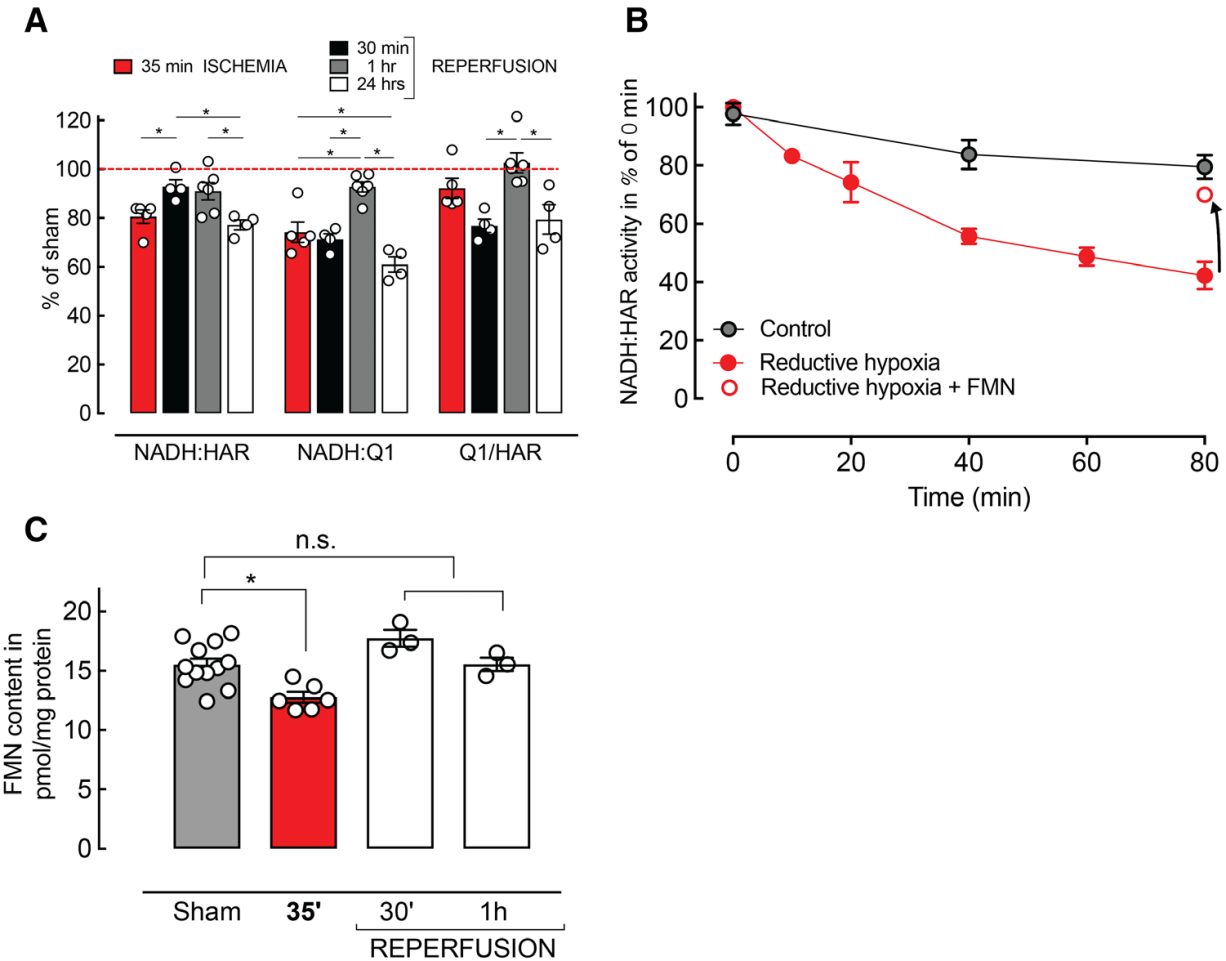
Complex I (C-I) impairment is associated with the multiphasic pattern of respiratory decline observed in ischemia/reperfusion (I/R). **A**, Overall activity of C-I at critical time points after I/R in mitochondrial membranes. **B**, In vitro time course of the reductive inactivation of NADH:hexaammineruthenium (HAR) reductase activity in mitochondrial membranes. Ischemic over-reduction of the ETC resulted in a decrease of the relative amount of C-I (red line) compared with control (black line). Addition of reduced FMN restored NADH:HAR reductase activity (arrow). **C**, Decrease of FMN in mitochondrial membranes obtained from the ischemic area after 35 minutes of middle cerebral artery occlusion (MCAO) compared with sham controls (n=6–12 per group; *P*=0.0048; ANOVA). n.s. indicates not signficant.

As shown in Figure 3A, 35 minutes ischemia resulted in a robust decline of NADH:Q_1_ reductase and NADH:HAR activity (74.2±4.2% and 80.5±2.7%, n=5 per group, respectively). A significant decrease in NADH:Q_1_ activity at 30 minutes (71.2±2.3%; n=4 per group), recovery at 1 hour (92.7±2.1%; n=6 per group), and another activity decline at 24 hours (58.9±3.3%; n=4 per group) of reperfusion was observed, confirming the results in whole tissue homogenates. Conversely, NADH:HAR activity showed a transient recovery at 30 minutes and 1 hour (92.9±2.9%; n=4 per group; 90.9±3.5%, n=6 per group, respectively) followed by a gradual decline at 24 hours after reoxygenation (77.2±2.0%; n=4 per group; Figure 3A).

The drop in physiological NADH:Q_1_ reductase activity could be explained by two fundamentally different mechanisms: a decline in C-I content or a decrease in the catalytic efficiency of C-I (number of NADH molecules oxidized by 1 enzyme molecule per minute). To estimate the relative catalytic efficiency of C-I, the ratio of NADH:Q_1_/NADH:HAR reductase (Q_1_/HAR) was calculated as previously described.^25^ No significant reduction in the catalytic efficiency of C-I after 35 minutes of ischemia (92.1±4.2%; *P*>0.05; n=5 per group) was observed. After reperfusion, a substantial decline in the efficiency of the enzyme was found at 30 minutes (76.8±2.8%; *P*<0.05; n=4 per group), followed by a complete recovery at 1 hour (102.6±4.0%; *P*>0.05; n=6 per group), and another decline at 24 hours (79.4±6.0%; *P*>0.05; n=4 per group) after I/R (Figure 3A). Note that although there was a significant decrease in NADH:Q1 and NADH:HAR reductase activities at 35 minutes ischemia, the Q1/HAR ratio did not change. This could be interpreted as decrease in the number of functional C-I molecules in the membrane with no change in the individual C-I enzyme catalytic efficiency (Q1/HAR).

### Functional Impairment C-I in Ischemia Is Because of a Loss of FMN

To further explore the transient decrease of NADH:HAR reductase after 35 minutes of ischemia, we performed in vitro experiments. Brain mitochondrial membranes from naive animals were incubated in conditions of metabolic reductive hypoxia,^26^ mimicking the over-reduction of the ETC in ischemia (Figure 3B). The first redox center of C-I, noncovalently bound FMN, is capable of dissociating from the enzyme.^27^ We found that incubation of mitochondrial membranes in reductive conditions resulted in a rapid decline of the HAR reductase activity over time, which seemed as a decrease of C-I content.

To confirm these in vitro findings, we determined the content of noncovalently bound FMN in mitochondrial membranes isolated at the critical time points after I/R. We found a significant decline of FMN content in the samples obtained after 35 minutes of ischemia (Figure 3C). This drop correlated with the decrease in NADH:HAR reductase activity in the same samples (Figure 3A, red bar) indicating ischemia-induced loss of FMN from the enzyme without a decrease in C-I content.

### Glutathione Improves C-I Dysfunction and Neurological Outcome After I/R

**Figure 4.**
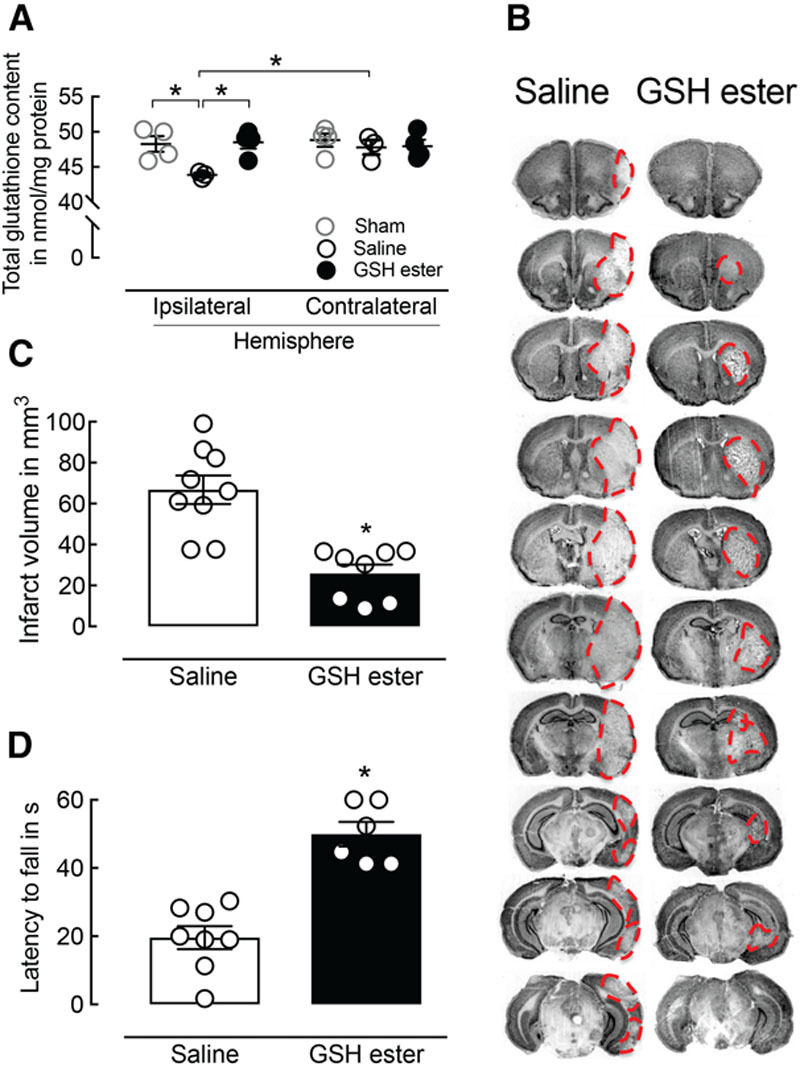
Restoration of total glutathione (GSH) tissue content attenuates ischemic injury and improves neurological outcome 72 hours after middle cerebral artery occlusion (MCAO). **A**, GSH ester treatment at the onset of reperfusion restored total GSH content in the ischemic brain area 30 minutes after reperfusion (n=3–4 per group; ANOVA). **B**, Reduced infarct volume in GSH-treated mice compared with controls 72 hours after MCAO (n=8–9 per group; *P*=0.0002; *t* test). **C**, Representative images of corresponding Nissl-stained brain sections of a GSH-treated mouse compared with control 3 days after MCAO. The red dashed line indicates the infarct area. **D**, GSH-treated mice show a significantly reduced motor impairment indicated by hanging wire test (6–8 per group; *P*<0.0001; *t* test).

Reperfusion-induced oxidative stress is one of the main contributors to tissue injury in I/R.^28,29^ Intracellular glutathione-dependent enzymatic systems regulate the thiol-based redox homeostasis and play a major role in the protection against oxidative stress. As shown in Figure 4A, I/R resulted in a significant decline of total glutathione content in the affected area in comparison to the contralateral hemisphere or sham. To test if reduced glutathione is able to confer a C-I–linked neuroprotection in vivo, we administered membrane-permeable glutathione-ethyl ester at the onset of reperfusion. Glutathione-ethyl ester restored total glutathione content in the ipsilateral hemisphere to control values (Figure 4A), indicating that it is able to penetrate into the brain tissue and interact with cellular glutathione pool. Furthermore, administration of glutathione-ethyl ester led to a 61% reduction in infarct volume (glutathione: 25.9±4.4 mm^3^ versus control: 66.8±7.0 mm^3^; *P*=0.0002; n=8–9 per group; Figure 4B and 4C), which correlated with decreased body weight loss (glutathione: 7.0±2.4% versus control: 19.8±2.9%; *P*=0.0037; n=8–9 per group; Figure IIA in the online-only Data Supplement). Overall functional outcome, assessed by the hanging wire test (Figure 4D) and modified Bederson score (Figure IIB in the online-only Data Supplement), was also improved compared with saline-treated controls.

### Glutathione Prevents Mitochondrial Dysfunction and C-I Activity Decline Early After I/R

**Figure 5.**
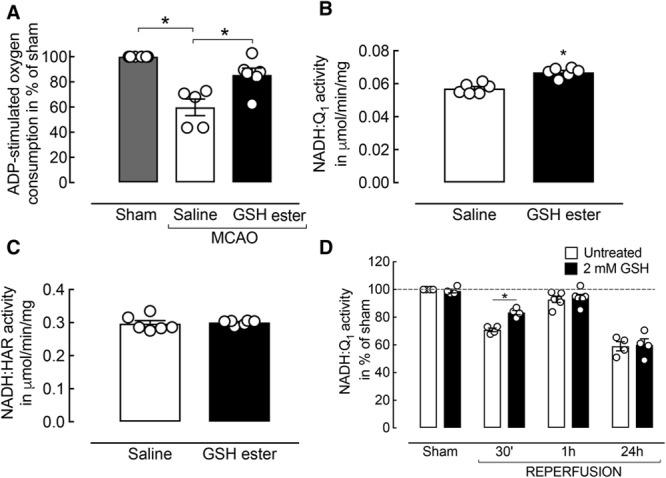
Glutathione (GSH) ester treatment improves complex I (C-I)–mediated bioenergetic failure early after reperfusion. **A**, GSH ester treatment ameliorates mitochondrial respiratory decline at 30 minutes of reperfusion (n=5–6 per group; *P*=0.03; Mann–Whitney *U* test). **B**, C-I activity is significantly improved in GSH-treated mice compared with controls (n=5–6 per group; *P*<0.05; Mann–Whitney *U* test). **C**, No change in the relative amount of C-I was observed. **D**, In vitro pre-incubation of whole tissue homogenates with GSH was able to partially recover ischemia/reperfusion (I/R)-induced C-I activity decline 30 minutes after reperfusion (n=4 per group; *P*=0.0009; *t* tests). GSH treatment did not affect C-I activity in sham, 1 or 24 hours after reperfusion. MCAO indicates middle cerebral artery occlusion.

To test the effect of glutathione-ethyl ester administration on mitochondrial function in vivo, we measured mitochondrial respiration 30 minutes after the onset of reperfusion comparing glutathione-treated mice and saline-treated controls. A significant increase in respiration was observed in tissue homogenates prepared from the ischemic area of glutathione-treated mice compared with saline-treated animals subjected to MCAO (*P*=0.03; n=5–6 per group; Figure 5A). These findings were associated with an increase in NADH:Q_1_ activity (*P*=0.0002; n=6 per group; Figure 5B). NADH:HAR activity was not affected (*P*>0.05; n=6 per group; Figure 5C) at 30 minutes of reperfusion.

Mitochondrial membranes isolated from the ischemic area of untreated animals at the critical time points after MCAO were preincubated ex vivo with thiol-reducing agent glutathione. NADH:Q_1_ and NADH:HAR activity were measured before and after glutathione incubation (Figure 5D). Pre-incubation with glutathione was able to recover NADH:Q_1_ activity in membranes obtained at 30 minutes of reperfusion (Figure 5D), indicating that reversible oxidation of C-I thiols is the underlying post-translational modification early after reperfusion. In contrast, glutathione treatment did not affect NADH:Q_1_ activity at 24 hours of reperfusion, pointing to an irreversible decline of C-I catalytic efficiency at later time points.

## Discussion

**Figure 6.**
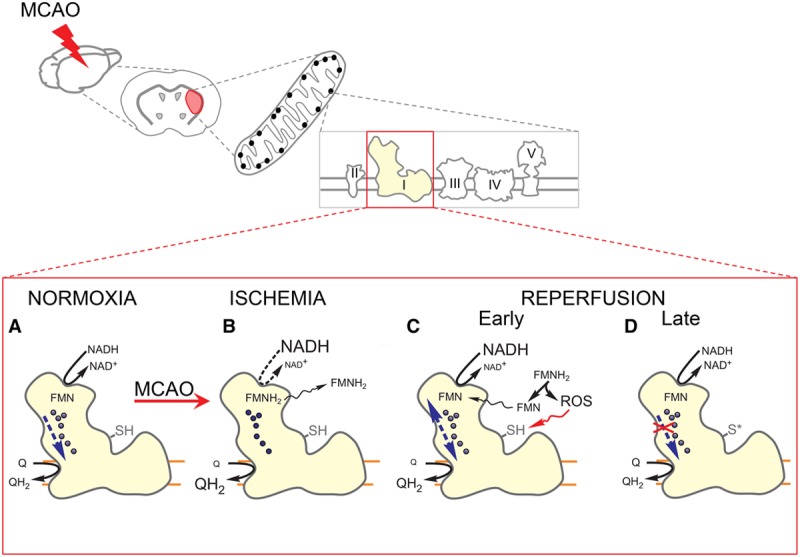
Proposed biochemical mechanisms contributing to complex I (C-I)–mediated energy failure in brain ischemia/reperfusion (I/R). **A**, Electron transfer within C-I during normoxia. **B**, Ischemic over-reduction of the ETC results in a reduction of C-I FMN. Reduced flavin (FMNH_2_) loses the affinity for its binding site and dissociates from the enzyme. **C**, Reflow-induced reoxidation of FMNH_2_ by molecular oxygen is associated with the generation of reactive oxygen species (ROS) and likely contributes to oxidative stress in the mitochondrial matrix. **D**, Recovery of C-I function at the early stage of reperfusion is followed by oxidation of critical C-I thiol residues (-SH) at later stages. MCAO indicates middle cerebral artery occlusion.

In the present study, we established a spatiotemporal profile of biochemical mechanisms contributing to the evolution of mitochondrial bioenergetic failure in I/R using a mouse model of transient MCAO. Using brain homogenates, we demonstrate an I/R-induced, multiphasic pattern of mitochondrial respiratory dysfunction in the brain, which to our knowledge has not been described before (Figure 6). We observed an initial decline in respiration after 35 minutes of ischemia, which is in agreement with previously published studies.^28,30^ The rapid partial recovery of mitochondrial respiration after 10 minutes of reperfusion followed by a first reflow-induced respiratory decline at 30 minutes of reoxygenation has never been reported. This decline in tissue respiration was followed by an almost full recovery at 1 hour with a slow decrease at later reperfusion time points (4–24 hours). In the samples from all time points, citrate synthase activity was similar to the sham controls, indicating preservation of mitochondrial mass, for 24 hours post-ischemia.

The rate of mitochondrial respiration can be used as a predictor of tissue survival after I/R.^31^ Our tissue preparations from the MCA area include a mixture of different brain cells so the observed changes in mitochondrial respiration cannot be exclusively attributed to only one cell type. Several publications have described a glia/neuron ratio of 0.4 to 0.35 in mouse brain,^32,33^ suggesting that neuronal mitochondria may contribute the major fraction of respiratory activity in brain homogenates.

We identified C-I as the key respiratory enzyme responsible for the multiphasic pattern of mitochondrial dysfunction in I/R. C-I has a high degree of flux control over oxidative phosphorylation and is considered to be the rate-limiting component of NADH oxidase activity within the ETC.^34^ Supporting our results, a comparable pattern of rotenone-sensitive C-I activity decline within 4 hours after reperfusion has been reported previously.^35^

The observed progressive decline in the enzymatic activity of C-IV after I/R injury is likely because of a different mechanism.^11^ C-II and C-III were not significantly affected in I/R, which is in agreement with previous in vivo studies investigating mitochondrial membrane complexes in stroke.^7,28,34,35^

Inhibition of NAD^+^-dependent respiration after ischemia has been observed in many stroke studies,^7,13,28,34,35^ but the mechanism was never established. Our results strongly suggest that ischemia induces a reversible release of FMN from C-I that caused the robust decrease of enzyme activity, which was rapidly restored within 10 minutes of reflow. C-I contains 1 molecule of noncovalently bound FMN per molecule of the enzyme,^36^ and it is the main source of membrane-associated flavin in mitochondria.^37^ FMN release is likely to occur in ischemia because of complex I over-reduction via reverse electron transfer.^38^ Reductive dissociation of C-I FMN has been reported in vitro^27,38^ but has not been shown in physiological settings. The release of a significant amount of reduced FMN (30–40 µmol/L) to the mitochondrial matrix is potentially harmful for the cell. On reperfusion, reduced FMN can be quickly reoxidized by oxygen,^39^ generating an equimolar amount of H_2_O_2_ in the matrix and significantly contributing to I/R-induced oxidative stress and tissue injury.

Mitochondrial function depends strongly on the maintenance of a cellular redox balance. Reperfusion triggers a burst of reactive oxygen species formation directly damaging cells via several different mechanisms.^40^ A critical component in the mitochondrial antioxidant defense system is endogenous glutathione. Reduced glutathione prevents or repairs oxidative damage generated by reactive oxygen species. Glutathione homeostasis is severely affected after I/R, therefore, making protein thiols a major target of oxidative damage.^41–43^ Mitochondrial respiratory enzymes are particularly susceptible to reactive oxygen species-mediated modulation of the thiol redox systems.^44,45^

We demonstrate that restoring total glutathione levels in the ischemic area at the onset of reperfusion is associated with protection of mitochondrial C-I activity and a robust neuroprotective effect. This is in agreement with previous studies showing a cytoprotective action of membrane-permeable thiol antioxidants against I/R-induced brain injury,^46–48^ but the mechanisms were not completely understood. Here, we presented evidence suggesting a reversible oxidation of critical thiols of C-I early after reperfusion, which is associated with a significant decrease in the enzymatic activity. Reconstitution of glutathione levels in vivo prevents mitochondrial bioenergetic dysfunction and C-I activity decline at 30 minutes of reperfusion. It should be noted that, in addition to protecting mitochondrial C-I, the antioxidant action of glutathione could also have beneficial impact through other cellular pathways, including inhibition of apoptosis^48^ and prevention of cytokine release.^46^ The ex vivo treatment of post-I/R mitochondrial membranes with glutathione recovered C-I activity at early, but not at late time points after reperfusion. Our data suggest that early reversible post-translational modifications of C-I are followed by an irreversible enzyme damage. On the basis of the neuroprotection of glutathione-ethyl ester and its positive effect on mitochondrial bioenergetics at 30 minutes of reperfusion, it is fair to speculate that this time point is particularly critical for the evolution of tissue infarction in our I/R model.

## Conclusions

We provide the first evidence that focal cerebral ischemia induces a C-I–mediated pattern of mitochondrial respiratory decline early after I/R. The ischemia-induced impairment of C-I activity is because of the reversible dissociation of reduced flavin from the enzyme (Figure 6). Because FMNH_2_ is a strong reactive oxygen species generator, this might be an important mechanism for the development of transient oxidative stress after reintroduction of oxygen on reperfusion.

Administration of ethyl ester of glutathione at the onset of reperfusion reduces infarction volume by 61% and improves neurological outcomes. This neuroprotective effect is associated with an increase of mitochondrial respiration and C-I activity. Thus, we conclude that reperfusion-induced C-I decline at 30 minutes after reperfusion is the result of a reversible modification of critical thiols of the enzyme. These findings indicate that preventing oxidative thiol modification of ETC early after the onset of reperfusion may be a viable approach to ameliorate mitochondrial dysfunction after I/R injury, ultimately reducing brain damage after stroke.

## Sources of Funding

This study was supported by Medical Research Council UK grant MR/L007339/1 (Dr Galkin) and National Institutes of Health grants R01NS34179 (Dr Iadecola) and R01NS095692 (Drs Manfredi and Iadecola). Dr Kahl was recipient of a postdoctoral research grant from the Deutsche Forschungsgemeinschaft (KA3810/1-1).

## Disclosures

None.

## Supplementary Material
