# Supplementary material for: Critical Role of Flavin and Glutathione in Complex I–Mediated Bioenergetic Failure in Brain Ischemia/Reperfusion Injury
Source: Stroke. 2018 Apr 11;49(5):1223–31. doi: 10.1161/STROKEAHA.117.019687 (PMC5916474; doi:10.1161/STROKEAHA.117.019687)
Supplement: Supplementary file 1 [file str-49-1223-s001.pdf]

ONLINE SUPPLEMENT

Critical role of flavin and glutathione in complex I-mediated bioenergetic failure in brain ischemia/reperfusion injury

Anja Kahl[1]*, Anna Stepanova[1,2]*, Csaba Konrad[1], Corey Anderson[1], Giovanni Manfredi[1], Ping Zhou[1], Costantino Iadecola[1] and Alexander Galkin[1,2]

[1]Feil Family Brain and Mind Research Institute, Weill Cornell Medicine, New York, USA

[2]School of Biological Sciences, Queen's University Belfast, UK

*These authors contributed equally to this work.

Material & Methods

Experimental animals

Experiments were performed in 7-9 week-old male C57Bl/6J mice (average weight: 23.2±1.1g, Jackson Laboratory) housed in an environmentally controlled room with a 12-hour light/dark cycle and fed a standard chow diet containing 13.2% fat, 24.6% protein and 62.1% carbohydrate (kcal/100 kcal) (#5053, LabDiet) with food and water ad libitum.

Middle Cerebral Artery occlusion model

Transient focal cerebral ischemia was induced in 7-9 week-old male mice using an intraluminal filament model of MCAO.[2] Briefly, mice were anesthetized with 1.5-2.0% isoflurane and rectal temperature was maintained at 37.3±0.3°C using a heating pad (TC-1000, CWE Inc.) during the surgical procedure and in the recovery period until the animals regained full consciousness.

A heat-blunted suture (6-0 suture) was inserted via the right external carotid artery until it obstructed the proximal part of the MCA and the common carotid artery simultaneously ligated for the duration of the ischemic period (35 min). Relative cerebral blood flow (CBF) was measured with transcranial laser Doppler flowmetry (Periflux System 5010, Perimed) in the center of the ischemic territory (coordinates: 2 mm posterior, 5 mm lateral to bregma). After 35 min, the filament was retracted and CBF reestablished. Only animals that exhibited a reduction in CBF of >85% during MCAO and in which CBF recovered by >80% after 10 min of reperfusion were included in the study.

Study timeline and experimental groups

The study was conducted between June 2015 till May 2017 and a total number of 151 animals were used. Overall 7 mice had to be excluded due to insufficient reperfusion (< 80% of relative baseline CBF), 1 mouse died before its 24 hour time-point and 3 mice had to be sacrificed due to bad physical condition before 3 days post-MCAO in the saline-treated control group.

Animals were randomly assigned to sham-operated and MCAO group or GSH ethyl ester treatment and saline treatment group, respectively. A consistent anesthesia time of 70 min was used for each animal in the MCAO and sham-operated group. For the 35 min ischemia only experimental group, the anesthesia time was adjusted to 30 min. This means that after the filament was inserted the CBF was only monitored for 10 min, until the incision site sutured and the animals put back into a preheated recovery cage. A separate corresponding sham served as a control for the ischemia only group.

Motor function testing

To detect a functional impairment three days post-MCAO, we used well-established motor function tests.[3, 4] The hanging wire test was selected to measure differences in grip strength, balance and endurance and the modified Bederson score was used to determine overall

functional outcome after MCAO.

Measurement of infarct volume

Infarct volume was measured in Nissl stained coronal brain sections (thickness: 30 µm; interval: 600 µm) throughout the infarcted territory (MCID, Imaging Research, UK) as described previously.[2] Post-ischemic edema was corrected by quantifying the difference in brain volume between the ischemic hemisphere and the contralateral side according to the method described previously.[2,5]

Preparation of brain homogenate and mitochondrial isolation

Following an MCAO period of 35 min only or including a recirculation period of 10, 30 min or 1, 2, 4, 6, 12, 24 h the animals were decapitated and membrane preparation isolated. The brain sample was homogenized in ice-cold isolation buffer (in mmol/L: 210 mannitol, 70 sucrose, 1 EGTA, 5 HEPES, pH 7.4) with 80 strokes of Dounce homogenizer. The homogenate was centrifuged at 1,000 × g for 5 min at 4°C and the supernatant was collected and used for analysis. For isolation of mitochondria, obtained homogenate was centrifugated for 15 min at 20,000 ×g. The resulting membrane pellet was rinsed twice with (in mmol/L) 250 sucrose, 50 Tris-HCl (pH 7.5), 0.2 EDTA medium and subsequently resuspended in the same medium. Frozen aliquots were stored at -80°C until use. Protein content was determined by BCA assay (Sigma) with 0.1% deoxycholate for solubilisation of mitochondrial membranes.

Mitochondrial respiration measurements

Mitochondrial respiration in homogenates (0.3±0.06 mg protein) was measured at 37°C in a 2 ml oxygen chamber (Oxygraph-2k, Oroboros Instruments, Innsbruck, Austria). Experimental buffer (in mmol/L: 225 mannitol, 70 sucrose, 5 HEPES, 4 K_2HPO_4, pH 7.4) contained C-I substrates (5 mmol/L glutamate and 2 mmol/L malate) as well as 1 mmol/L $MgCl_2$ and 0.1 mmol/L EGTA. Measurements were recorded for steady-state basal oxygen consumption and following the addition of 0.2 mmol/L ADP (state 3 respiration), 1 µmol/L oligomycin (state 2 respiration) and 1 mmol/L KCN respectively. The respiratory control ratio (RCR) was calculated as the ratio of ADP-stimulated (state 3) to oligomycin-treated respiration corrected for cyanide-insensitive activity. 100% corresponds to 25.6±1.4 nmol O_2×min^{-1}×mg^{-1}.

Enzyme activity measurements in mitochondrial membranes

All activities were measured spectrophotometrically using Molecular Devices SpectraMax plate reader or spectrophotometer Perkin Elmer Lambda 35 in 0.2 or 2 ml of the assay buffer respectively. NADH-dependent enzymatic activities were assayed in SET media (in mmol/L: 50 Tris-Cl pH 7.5, 250 sucrose, and 0.2 EDTA) with 30 µg/mL alamethicin, 1 mmol/L $MgCl_2$. Succinate:cytochrome *c* reductase was assayed in KCl media (in mmol/L: 125 KCl, 14 NaCl, 0.2 EGTA, 20 HEPES-Tris, pH 7.2), and succinate:DCIP reductase activity of complex II was assayed in 20 mmol/L HEPES buffer pH 7.8.

Rotenone-sensitive activities of complex I were assayed as a decrease in absorption at 340 nm (ε_{340nm} = 6.22 L·mmol^{-1}cm^{-1}) with 150 µmol/L: NADH in SET media supplemented with 15 µmol/L: cytochrome *c* for NADH-oxidase or with 1mmol/L KCN and 50 µmol/L Q_1 for NADH:Q_1 reductase activity. 100% corresponds to 206.0±11.2 and 45.4±2.6 nmol NADH×min^{-1}×mg^{-1} for NADH-oxidase and NADH:Q_1 reductase. Only rotenone sensitive part of activities was taken for the calculations. NADH:hexammineruthenium (HAR) oxidoreductase reductase [6, 7] was assayed in the SET medium supplemented with 1 mmol/L cyanide and 1 mmol/L HAR and 0.025% dodecylmaltoside.

Complex II-dependent activities were measured after malonate activation of the sample as described in detail in [8, 9]. Succinate:DCIP activity was recorded as a decrease in absorption at 600 nm (ε_{600nm} = 21 L·mmol^{-1}cm^{-1}) in HEPES buffer containing 15 mmol/L succinate, 100 µmol/L Q_1, 80 µmol/L DCIP. 100% corresponds to 26.1±1.7 nmol×min^{-1}×mg^{-1}.

Antimycin A sensitive succinate:cytochrome *c* reductase was measured as an increase in cytochrome *c* absorption at 550 nm (ε_{550nm} = 21.5 L·mmol^{-1}cm^{-1}) in KCl media, containing 15 mM succinate, 50 µmol/L cytochrome *c*, and 1 mmol/L KCN. 100% corresponds to 7.5±0.8 nmol×min^{-1}×mg^{-1}

Complex IV activity was measured as oxidation of 50 µmol/L ferrocytochrome *c* at 550 nm ($\varepsilon_{550\ nm}$ = 21.0 L·mmol^{-1}cm^{-1}) in 1 mL of SET/2 buffer supplemented with 0.025% dodecylmaltoside. Ferrocytochrome *c* oxidase activity was fully sensitive to cyanide. 100% corresponds to 658.3±28.7 nmol×min^{-1}×mg^{-1}.

Following protein concentration was used for the measurements of the activities (in µg/ml): NADH oxidase, 25-50; NADH:HAR reductase, 15-25; NADH:Q_1 reductase, 50-100; succinate DCIP reductase, 25-75; succinate:cytochrome c reductase, 100-150.

GSH treatment of the membranes was performed as described earlier with minor modifications.[10] To obtain mitochondrial membranes, the homogenates were centrifuged at 20,000 x g for 15 min at 4°C and washed twice with the homogenization buffer. Membranes (10-20 mg/mL of protein) were incubated with 2 mmol/L GSH for 5 minutes in SET media supplemented with 30 µg/mL alamethicin, 1 mmol/L $MgCl_2$, 60 µmol/L Q_1 and 1 mmol/L KCN. NADH:Q_1 activity was recorded after the addition of 100 µmol/L NADH. NADH:HAR reductase was measured in a similar way.

Citrate synthase activity assay

Citrate synthase activity was measured as described earlier,[11] with minor modifications. Briefly, frozen-thawed whole tissue homogenates were diluted in 0.01% Triton, and 10 µl of each sample (30 µg protein) were loaded into a well of 96-well plate. Assay media (0.2 mL) contained 20 mmol/L HEPES buffer pH 7.8, 0.1 mmol/L DTNB, 0.4 mmol/L Ac-CoA, 0.4 mmol/L OAA and 30 µg protein. The activity was determined at 412 nm ($\varepsilon_{412\ nm}$ = 14.2 L×mmol^{-1}cm^{-1}) using a plate reader SpectraMax M5 (Molecular Devices). 100% corresponds to 37.8±3.2 nmol×min^{-1}×mg^{-1} protein^{-1}.

Determination of membrane bound FMN

Approximately 100 µg of mitochondria membrane protein was diluted with 20 mmol/L HEPES, pH 7.4, to 1 mg/mL and mixed with an equal volume of 15% TCA for deproteination. After incubation on ice for 10 minutes, protein precipitate was removed by centrifugation at 6,600 x g for 10 min. To neutralize the supernatant, 10 µL of 1 mol/L HEPES pH 7.4, and 20 µL of 5 mol/L KOH were added per 200 µL of the supernatant as previously described with minor modifications.[12]

Determination of acid-extractable flavin was performed according to Faeder et al.,[13] with slight modifications. Fluorescence emission was measured at 525 nm, with the excitation at 470 nm (Hitachi F-7000 fluorospectrophotometer) at two different pH (7.6 and 2.3) in 0.1 mol/L phosphate buffer containing 0.1 mmol/L EDTA. Freshly prepared standard solutions of FMN and FAD with known concentration were used for calibration.

Evaluation of total glutathione content

After administration of GSH ester, the content of total glutathione (GSH and GSSG) was determined using a Glutathione Assay Kit (703002, Cayman). Briefly, pieces of frozen tissue were homogenized in ten volumes of 50 mmol/L MES buffer pH 6.5, 1 mmol/L EDTA with tissue disruptor (Dremel, Tissue-Tearor). Further steps were performed according to the manufacturer instructions. For the GSSG measurement, samples were incubated with 10 µmol/L 2-vinylpyridine for an hour at room temperature.

Western Blot

Immunoblot analyses were performed as previously described, [9] using total OXPHOS rodent primary antibody cocktail (ab110413, Abcam, diluted 1:1,000 in 3% BSA in TBS+0.01% Triton-X containing five different antibodies against the all OXPHOS complexes. Tim23 staining (1:1,000, BD Transduction Lab) was used to monitor equal gel loading.

Experimental design and statistical analysis

GraphPad Prism software (version 7.0, GraphPad Software) was used for all statistical analysis. Data are expressed as mean±SEM. Intergroup differences between two groups were analyzed by unpaired Student's *t* test or non-parametric Mann-Whitney *U* test, as appropriate. If more than two groups were analyzed and compared to sham, one-way ANOVA with Dunnett's multiple comparisons test or Kruskal Wallis test with Dunn's multiple comparisons test was used. Ordinary one-way ANOVA with Tukey´s multiple comparisons test was performed if multiple groups were compared to each other. As for the *in vitro* experiment with GSH treatment of the mitochondrial membranes, differences were analyzed by paired Student´s *t* test. Differences were considered statistically significant for $*p<0.05$. For the *in vivo* GSH study the number of experimental animals required to detect a standardized effect size > 0.25 was calculated by a priori power analysis with the following assumptions: power = 0.8 and $\alpha = 0.05$, SD 20% of the mean (GPower 3.1 software).

Figure I

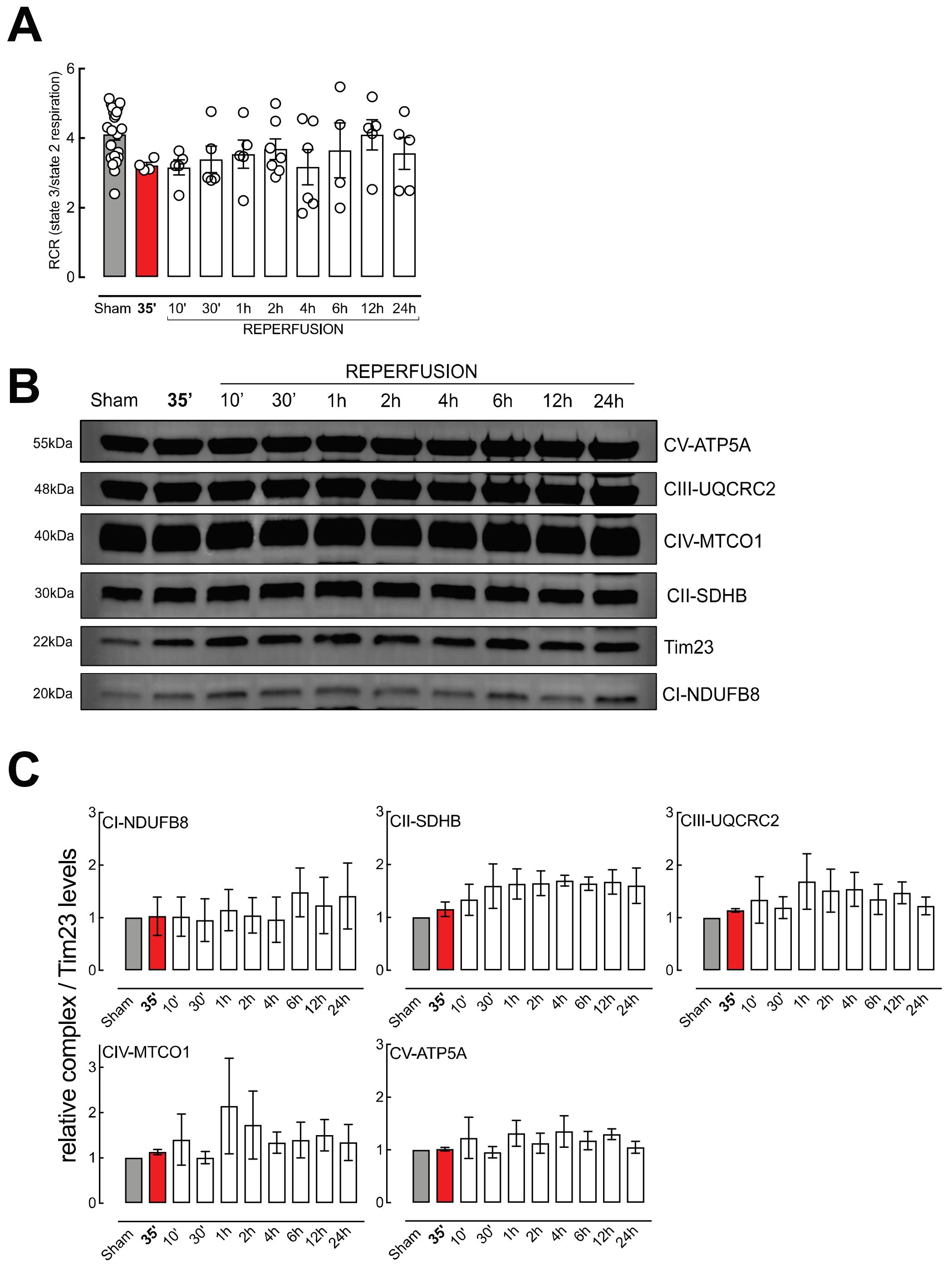

Figure I. Protein levels of individual respiratory chain complexes and respiratory control did not change after focal cerebral ischemia. (A) No changes in respiratory control ratio (RCR), a parameter reflecting the integrity of the mitochondrial inner membrane, did not change at any time point during the first 24 h of reperfusion ($p>0.05$; n=4-7 mice per group) compared to sham-operated controls (n=19). (B,C) Representative immunoblot analysis including abundance quantification of mitochondrial respiratory chain complex subunits from whole tissue homogenates after 35 min ischemia and the first 24 h after reperfusion. The band intensities were normalized to the mitochondrial membrane protein Tim23, $p>0.05$, n=3.

Figure II

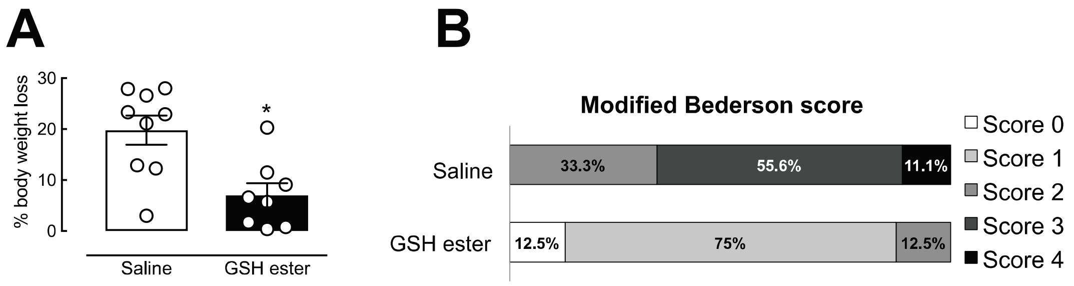

Figure II. GSH ester treated mice show improved overall outcome 72 h after focal cerebral ischemia. (A) Body weight loss analysis of GSH ester and control mice 72 h after MCAO (n= 8-9 mice per group, p=0.0037, unpaired *t* test). (B) GSH treated mice show an improved modified Bederson score; n= 8-9 mice per group 72 h after MCAO.

Supplemental References:

1. Kilkenny C, Browne WJ, Cuthill IC, Emerson M, Altman DG. Improving bioscience research reporting: The ARRIVE guidelines for reporting animal research. *PLoS Biol*. 2010;8:e1000412
2. Jackman K, Kunz A, Iadecola C. Modeling focal cerebral ischemia *in vivo*. *Methods Mol Biol*. 2011;793:195-209
3. Abe T, Shimamura M, Jackman K, Kurinami H, Anrather J, Zhou P, et al. Key role of cd36 in toll-like receptor 2 signaling in cerebral ischemia. *Stroke*. 2010;41:898-904
4. Balkaya M, Krober JM, Rex A, Endres M. Assessing post-stroke behavior in mouse models of focal ischemia. *J Cereb Blood Flow Metab*. 2013;33:330-338
5. Lin TN, He YY, Wu G, Khan M, Hsu CY. Effect of brain edema on infarct volume in a focal cerebral ischemia model in rats. *Stroke*. 1993;24:117-121
6. Sled VD, Vinogradov AD. Kinetics of the mitochondrial NADH-ubiquinone oxidoreductase interaction with hexammineruthenium (iii). *Biochim. Biophys. Acta*. 1993;1141:262-268
7. Birrell JA, Yakovlev G, Hirst J. Reactions of the flavin mononucleotide in complex I: A combined mechanism describes nadh oxidation coupled to the reduction of $APAD^+$, ferricyanide, or molecular oxygen. *Biochemistry*. 2009;48:12005-12013
8. Ackrell BA, Kearney EB, Singer TP. Mammalian succinate dehydrogenase. *Methods Enzymol*. 1978;53:466-483
9. Stepanova A, Shurubor Y, Valsecchi F, Manfredi G, Galkin A. Differential susceptibility of mitochondrial complex ii to inhibition by oxaloacetate in brain and heart. *Biochim. Biophys. Acta*. 2016;1857:1561-1568
10. Galkin A, Moncada S. S-nitrosation of mitochondrial complex i depends on its structural conformation. *J. Biol. Chem.* 2007;282:37448-37453
11. Srere PA. Citrate synthase. *Methods Enzymol.* 1969;13:3-11
12. Merola AJ, Coleman R, Hansen R. Flavin mononucleotide: The acid-extractable flavin of the DPNH-coenzyme q reductase of the respiratory chain. *Biochim. Biophys. Acta*. 1963;73:638-640
13. Faeder EJ, Siegel LM. A rapid micromethod for determination of FMN and FAD in mixtures. *Anal Biochem*. 1973;53:332-336
