# Supplementary material for: Critical Role of Flavin and Glutathione in Complex I–Mediated Bioenergetic Failure in Brain Ischemia/Reperfusion Injury
Source: Stroke. 2018 Apr 11;49(5):1223–31. doi: 10.1161/STROKEAHA.117.019687 (PMC5916474; doi:10.1161/STROKEAHA.117.019687)
Supplement: Supplementary file 2 [file str-49-1223-s002.pdf]

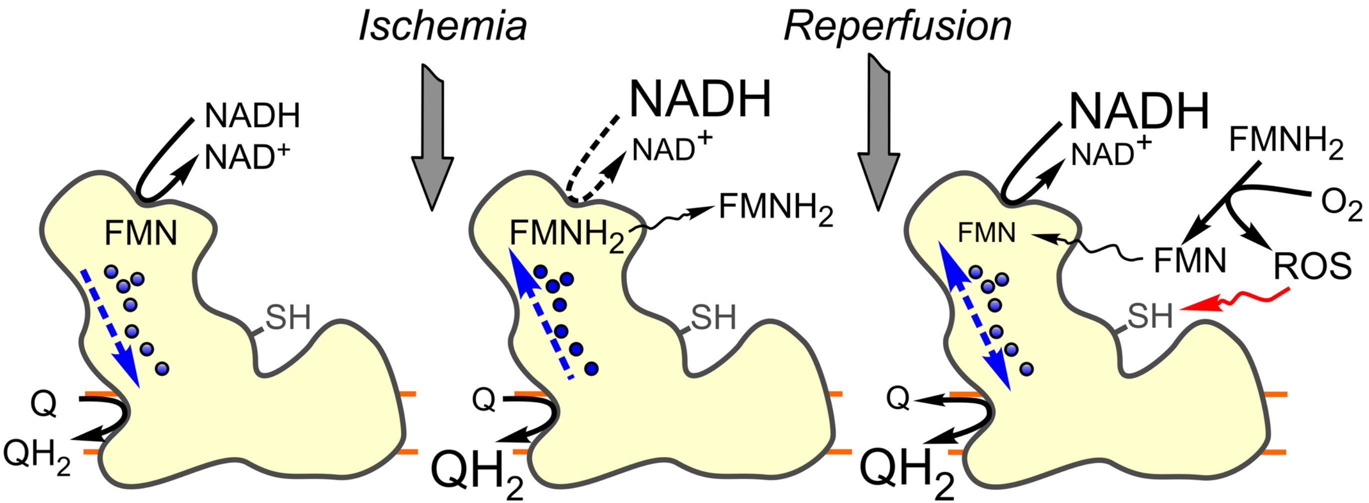


Over-reduction of mitochondrial complex I during brain ischemia results in a dissociation of reduced flavin ($FMNH_2$) from the enzyme. Reperfusion-induced reoxidation of $FMNH_2$ by molecular oxygen is associated with generation of ROS and contributes to oxidative stress in the mitochondrial matrix. Recovery of complex I function at the early stage of reperfusion is followed by oxidation of critical thiol residue(s) at later stages.
